# Insight into blood pressure targets for universal coverage of hypertension services in Iran: the 2017 ACC/AHA versus JNC 8 hypertension guidelines

**DOI:** 10.1186/s12889-020-8450-1

**Published:** 2020-03-17

**Authors:** Mahdi Mahdavi, Mahboubeh Parsaeian, Bahram Mohajer, Mitra Modirian, Naser Ahmadi, Moein Yoosefi, Parinaz Mehdipour, Shirin Djalalinia, Nazila Rezaei, Rosa Haghshenas, Forough Pazhuheian, Zahra Madadi, Mahdi Sabooni, Farideh Razi, Siamak Mirab Samiee, Farshad Farzadfar

**Affiliations:** 1 National Institute of Health Research, Tehran University of Medical Sciences, Tehran, Iran; 2 Erasmus School of Health Policy and Management, Erasmus University Rotterdam, Rotterdam, The Netherlands; 3 Department of Epidemiology and Biostatistics, School of Public Health, Tehran University of Medical Sciences, Tehran, Iran; 4 Non-Communicable Diseases Research Center, Endocrinology and Metabolism Population Sciences Institute, Tehran University of Medical Sciences, Tehran, Iran; 5 Deputy of Research and Technology, Ministry of Health and Medical Education, Tehran, Iran; 6 Endocrinology and Metabolism Research Center, Endocrinology and Metabolism Clinical Sciences Institute, Tehran University of Medical Sciences, Tehran, Iran; 7 Reference Health Laboratory, Ministry of Health and Medical Education, Tehran, Iran; 8 Diabetes Research Center, Endocrinology and Metabolism Clinical Sciences Institute, Tehran University of Medical Sciences, Tehran, Iran

**Keywords:** Hypertension, JNC8 hypertension guideline, 2017 ACC/AHA hypertension guideline, Effective coverage, Prevalence, Awareness, Treatment, Control, Iran

## Abstract

**Background:**

We compared the prevalence, awareness, treatment, and control of hypertension in Iran based on two hypertension guidelines; the 2017 ACC/AHA –with an aggressive blood pressure target of 130/80 mmHg- and the commonly used JNC8 guideline cut-off of 140/90 mmHg. We shed light on the implications of the 2017 ACC/AHA for population subgroups and high-risk individuals who were eligible for non-pharmacologic and pharmacologic therapies.

**Methods:**

Data was obtained from the Iran national STEPS 2016 study. Participants included 27,738 adults aged ≥25 years as a representative sample of Iranians. Regression models of survey design were used to examine the determinants of prevalence, awareness, treatment, and control of hypertension.

**Results:**

The prevalence of hypertension based on JNC8 was 29.9% (95% CI: 29.2–30.6), which soared to 53.7% (52.9–54.4) based on the 2017 ACC/AHA. The percentage of awareness, treatment, and control were 59.2% (58.0–60.3), 80.2% (78.9–81.4), and 39.1% (37.4–40.7) based on JNC8, which dropped to 37.1% (36.2–38.0), 71.3% (69.9–72.7), and 19.6% (18.3–21.0), respectively, by applying the 2017 ACC/AHA. Based on the new guideline, adults aged 25–34 years had the largest increase in prevalence (from 7.3 to 30.7%). They also had the lowest awareness and treatment rate, contrary to the highest control rate (36.5%) between age groups. Compared with JNC8, based on the 2017 ACC/AHA, 24, 15, 17, and 11% more individuals with dyslipidaemia, high triglycerides, diabetes, and cardiovascular disease events, respectively, fell into the hypertensive category. Yet, based on the 2017 ACC/AHA, 68.2% of individuals falling into the hypertensive category were eligible for receiving pharmacologic therapy (versus 95.7% in JNC8). LDL cholesterol< 130 mg/dL, sufficient physical activity (Metabolic Equivalents≥600/week), and Body Mass Index were found to change blood pressure by − 3.56(− 4.38, − 2.74), − 2.04(− 2.58, − 1.50), and 0.48(0.42, 0.53) mmHg, respectively.

**Conclusions:**

Switching from JNC8 to 2017 ACC/AHA sharply increased the prevalence and drastically decreased the awareness, treatment, and control in Iran. Based on the 2017 ACC/AHA, more young adults and those with chronic comorbidities fell into the hypertensive category; these individuals might benefit from earlier interventions such as lifestyle modifications. The low control rate among individuals receiving treatment warrants a critical review of hypertension services.

## Background

Hypertension (HTN) is the leading modifiable risk factor for premature morbidity and mortality in the world and Iran. The prevalence of HTN is rising globally [1]. In 2000, 26.4% of the world’s adults had HTN, which is expected to reach 29.2% by 2025 [2]. Among Iranians aged 25–70 years, 24.1% were living with HTN in 2011 [3]. The fact that a large proportion of the population is living with HTN and its costly comorbidities make it a health priority and a tracer for measuring progress towards Universal Health Coverage (UHC).

The definition of hypertension, which determines a cut-off for hypertension diagnosis, directly affects the estimates made for the UHC of hypertension i.e. prevalence, awareness, treatment and control, and subsequently, the treatment costs incurred by health systems for hypertension control. Following the release of the 2017 ACC/AHA Guideline for the Prevention, Detection, Evaluation, and Management of High Blood Pressure in Adults with the cut-off of 130/80 mmHg [4], a reasonable blood pressure (BP) target for the effective coverage of hypertension has become a heated debate [5]. For several years, guidelines such as the JNC8 (with 140/90 mmHg as its cut-off for hypertension) were used to determine the prevalence, awareness, treatment, and control of hypertension [6].

Studies have shown that switching from JNC8 to the 2017 ACC/AHA increases the prevalence of hypertension [7, 8]. However, evidence on the implications of the 2017 ACC/AHA guideline on the awareness, treatment, and control of hypertension is under-developed [9]. Despite the potential health benefits of the 2017 ACC/AHA guideline [7, 8], adopting this guideline to enhance hypertension control in low- and middle- income countries (LMICs) is under question – given its economic impacts [10]. According to Watkins, the burden of the possibly higher numbers of individuals that shift from the ‘elevated’ and ‘prehypertensive’ into the ‘hypertensive’ categories (based on the 2017 ACC/AHA guideline) is hardly bearable by the already-overburdened health systems of LMICs [11]. This counterargument warrants more empirical findings per country in order to estimate the burden of embarking on the 2017 ACC/AHA hypertension guideline.

In this study, we estimated the prevalence, awareness, treatment, and control of HTN, based on the two ‘JNC8’ and ‘2017 ACC/AHA’ guidelines. We shed light on the implications (and benefits) of adopting an intensified blood pressure control recommended by the 2017 ACC/AHA, for different subgroups of Iranian populations and high-risk hypertensive adults using the 10-year atherosclerotic cardiovascular disease (ASCVD) risk score. We compared the proportion of hypertensive individuals eligible for pharmacologic therapy based on both guidelines and discussed the implications in terms of the potential costs imposed on the Iranian health system to provide treatment to adults eligible for pharmacologic therapy.

## Methods

### Research design

We used the data collected in the ‘Iran STEPS 2016’ study. The WHO STEPwise approach to Surveillance (STEPS) provided the grounds for conducting the Iran STEPS 2016 study [12]. The Iran STEPS 2016 study included a representative sample of the Iranian population from urban and rural areas of 30 provinces, which were selected based on a multistage random sampling method. All Iranians aged > 18 years who were living in Iran at the time of data collection were eligible for inclusion in the study. The original study questionnaire was constructed by the WHO STEPS. It was translated into Persian and was culturally adapted through learning from the application of the questionnaire in the earlier STEPS studies conducted in 2005, 2006, 2007, 2008, 2009, and 2011. During this development process, the consistency, validity, and reliability of the questionnaire were assessed. Data was collected by trained interviewers through in-person interviews. The methods employed in the Iran STEPS 2016 study which include details on the sampling design, the validity and reliability of the study questionnaire, the interview guide, and data collection methods are presented elsewhere [13]. The interview guide was not developed for the present study, but for the Iran STEPS 2016 study.

### Measures

#### Outcome definitions

The main outcomes consisted of prevalence, awareness, treatment, and control of HTN. We distinguished hypertension based on the JNC8 and the 2017 ACC/AHA guidelines. Based on JNC8, we considered individuals with systolic blood pressure (SBP) ≥140 mmHg or diastolic blood pressure (DBP) ≥90 mmHg as hypertensive; whereas, based on 2017 ACC/AHA, those with SBP ≥ 130 mmHg or DBP ≥ 80 mmHg [4] were considered hypertensive. Furthermore, the self-reported use of antihypertensive drugs in the last 2 weeks was considered as the presence of HTN for both definitions [14]. According to the WHO STEPS manual, trained personnel measured blood pressure on the right upper-arm three times, having had the participant rest for 5 min in a seated position [12]. An average of the last two measurements was considered as the blood pressure measure. Awareness was deemed to be present if an individual answered ‘Yes’ to the question ‘Have you ever been diagnosed with hypertension by a physician or a health professional?’ Treatment was defined as the self-reported use of antihypertensive drugs among aware individuals. Hypertension control referred to an average SBP < 130 & DBP < 80 mmHg based on the 2017 ACC/AHA and an average SBP < 140 & DBP < 90 mmHg based on the JNC8.

#### Covariates

Covariates included demographic, socio-economic status (SES), lifestyle, health insurance coverage, and cardiovascular disease (CVD) risk factors. Demographic factors included age, gender, marital status, and place of residence. Age groups consisted of 25–34, 35–44, 45–54, 55–64, 65–74, and 75+ years. Marital status included two groups; single/divorced/widow and married. SES comprised of wealth status and the years of schooling. Wealth status was measured by the wealth index [15] and was grouped into the poorest, poor, average, rich, and richest. Based on the years of schooling, participants were categorized into four groups; participants with no schooling, 1–6 years, 7–12 years, and higher than 12 years of schooling. Insurance coverage referred to basic and complementary health insurance. Basic health insurance refers to a minimum coverage of essential health services by public health insurance organizations. Complementary health insurance is a coverage policy provided by private insurers that pays for surcharges of medical services not covered by basic health insurance or services delivered by private providers [16].

Lifestyle factors consisted of smoking, alcohol consumption, intake of fruits and vegetables, salt intake, and physical activity. Smoking has a dichotomy of statuses: never-smoker/non-smoker and current daily cigarette smoker. Smoker referred to a person who smoked cigarettes on a daily basis at the time of the survey. Never-smoker/non-smoker referred to a person who had never smoked or had quitted smoking. Since evidence on the relationships between smoking and hypertension is controversial, we relied on [17] comparing the outcomes between former-smokers and never-smokers versus current-smokers. Furthermore, only 70 (0.26%) study participants reported using tobacco products other than cigarettes; whereas, 2911 (10.72%) study participants reported that they were current cigarette smokers. Consequently, smoking other tobacco products was not included in the analysis and our analysis focused on cigarette smoking. In terms of alcohol intake, we classified the participants into alcohol drinkers and non-drinkers. An alcohol drinker referred to a person who had consumed any type of alcohol product during the last 12 months before the time of the survey, regardless of the duration or frequency of consumption. Non-drinker referred to a participant who had consumed no alcohol during the same period of time. The intake of fruits and vegetables was estimated for 24 h (24-h). We considered five portions of fruits and vegetables, consisting of two portions of fruits and three portions of vegetables, as a sufficient daily intake based on the dietary guidelines [18]. One portion of fruits referred to 80 g of fruits. In order to make the portion size comprehensible to the study participants, ‘one medium-sized fruit, like a medium-sized apple, or ¼^th^ of a cup of dried fruits’ was considered one portion of fruits. One portion of vegetables equalled ‘one cup of raw leafy vegetables, like spinach, or half a cup of cooked vegetables’.

The 24-h salt intake was estimated from spot-urine samples using the Tanaka equation [19]:

$$ 2.54\div 1000\times 23\times 21.98\times {\left\{ spot\ sodium\;\left( mmol/l\right)/\left[ spot\ creatinine\;\left( mg/ dL\right)\times 10\right]\times \left[-2.04\times age\;(years)+14.89\times weight\;(kg)+16.14\times height\;(cm)-2244.45\right]\right\}}^{0.392} $$

All spot urine samples were collected in the morning between 8.00–10.00 a.m., and transferred to a central laboratory unit according to the 2016 STEPS study protocol [13]. The detailed methods and results of applying the Tanaka equation to the Iran STEPS 2016 data have been published elsewhere [20]. We analysed the relationships between salt intake and outcomes using salt intake as a continuous and dichotomized variable. Since only 2% of the study sample had a salt intake of < 5 g/day, we considered 10 g/day as the cut-off. The complementary analysis of relationships between daily salt intake and blood pressure among hypertensive, aware, treatment-receiving, and under-control individuals is presented in Additional File 1. Physical activity was measured using the WHO Global Physical Activity Questionnaire (GPAQ) version 2 with a cut-off of metabolic equivalents (METs) ≥ 600/week as sufficient [21]. Body Mass Index (BMI) had four levels; underweight (< 18.5 kg/m^2^), normal (18.5–24.9 km/m^2^), overweight (25.0–29.9 kg/m^2^), and obesity (≥30 kg/m^2^). CVD risk factors consisted of dyslipidaemia, high triglycerides, diabetes mellitus (DM), and self-reported history of CVDs, i.e. myocardial infarction and/or stroke [4]. Dyslipidaemia referred to either total cholesterol ≥200 mg/dL, high-density lipoprotein (HDL) cholesterol < 35 mg/dL, or low-density lipoprotein (LDL) cholesterol ≥130 mg/dL. High triglycerides referred to fasting triglycerides ≥200 mg/dL [22]. DM referred to HbA1c > 48 mmol/mol or fasting blood sugar (FBS) > 126 mg/dL or self-reported DM [23].

### Statistical analysis

We calculated the ratio and 95% confidence interval (95% CI) of prevalence, awareness, treatment, and control based on cut-offs recommended by the JNC8 and 2017 ACC/AHA. We constructed univariate and multiple logistic regression models to account for the effects of covariates on prevalence, awareness, treatment, and control based on the 2017 ACC/AHA only. We calculated the number and percentage of individuals eligible for pharmacologic therapy based on both JNC8 [6] and the 2017 ACC/AHA. The number of individuals eligible for nonpharmacologic therapy was only determined based on the 2017 ACC/AHA, which comprised elevated, HTN stage 1, and HTN stage 2 adults. Based on 2017 ACC/AHA, two groups were eligible for pharmacologic therapy: a) individuals with BP ≥140/90, and b) those with BP ≥130/80 who had 10-year atherosclerotic CVD (ASCVD) risk≥10%. The number of adults eligible for pharmacologic therapy was also determined based on JNC8 [6]. Among individuals with BP ≥ 120/80 mmHg, associations between blood pressure and lifestyle factors, weight, BMI, physical activity, intake of fruits and vegetables, 24-h intake of salt, LDL cholesterol, and alcohol consumption were tested.

Given the multistage clustering structure, a complex survey analysis was used to obtain summary measures and statistical models. We weighted samples according to the 2015 Iranian National Population Census. Logistic regression models with a survey design was used to analyse associations between the outcomes and covariates. We analysed the data using Stata 13 and R 3.4.1 statistical software programs.

## Results

The study sample included 27,738 participants who were aged ≥25 years. Of these, 573 (2%) were excluded from the analyses due to missing values of SBP or DBP measurements. In the end, 27,165 participants were considered for analysis, of whom about 70% were between 25 and 54 years old.

We found that adopting 2017 ACC/AHA markedly increased the prevalence. Based on JNC8, the prevalence was 29.9% (95% CI: 29.2–30.6), which soared to 53.7% (52.9–54.4), based on the 2017 ACC/AHA (Table S1). Likewise, the prevalence rate sharply increased by age from younger to older groups, reaching its peak at 82.4% among those ≥75 years old (Odds Ratio (OR): 7.97 [6.29–10.10]) (Table S1). Based on the 2017 ACC/AHA, the largest increase in prevalence was observed in the 25–34-year-old age group; the prevalence increased from 7.3% based on JNC8 to 30.7% based on the 2017 ACC/AHA.

The prevalence percentage was lower among females (OR: 0.74 [0.67–0.82]) and rural dwellers (OR: 0.90 [0.80–1.00]). Among the wealth groups, the richest group had the lowest prevalence (OR: 0.69 [0.58–0.82]). The prevalence also significantly decreased from 73.7% among illiterates to 44.2% among those with > 12 years of schooling (OR: 0.66 [0.55–0.79]). Prevalence significantly increased from normal BMI to overweight (OR: 1.60 [1.44–1.78]) and obese (OR: 2.22 [1.97–2.51]). It was significantly higher among those with dyslipidaemia (OR: 1.15 [1.05–1.26]), high triglycerides (OR: 1.31 [1.15–1.49]), DM (OR: 1.58 [1.37–1.82]), and CVD history (OR: 1.77 [1.28–2.45]). By lowering the blood pressure cut-off point by 10 mmHg to 130/80 mmHg, 24, 15, 17, and 11% more individuals with dyslipidaemia, high triglycerides, diabetes, and CVD events, respectively, fell into the hypertensive category. For instance, based on the JNC 8, 71.5% of individuals with previous CVD events were considered hypertensive, which increased to 82.5% based on the 2017 ACC/AHA (Table S1).

Based on the JNC8, 59.2% (58.0–60.3) of hypertensive individuals were aware; whereas, according to the 2017 ACC/AHA, 37.1% (36.2–38.0) were aware (Table S2). Based on the 2017 ACC/AHA guideline, awareness significantly increased by age, from 9.7% in the youngest to 67.9% in the oldest group (OR: 13.23 [9.54–18.37]). A larger proportion of females (45.1%) were aware (OR: 1.59 [1.39–1.81]) compared to males (28.9%). Awareness declined with increases in years of schooling, reaching its lowest among the well-educated group (OR: 0.58 [0.46–0.73]). Patients with a higher awareness were more likely to have complementary insurance coverage.

Awareness was significantly higher among the overweight (OR: 1.39 [1.19–1.62]) and obese (OR: 1.71 [1.44–2.03]) groups. Individuals with salt ≥10 g/day were less likely to be aware (OR: 0.87 [0.77–0.98]) and one gram/day salt decreased the odds of awareness (OR: 0.96 [0.94–0.99]) (see also Additional File 1). Awareness was significantly higher among individuals with DM (OR: 1.76 [1.52–2.05]) and a history of CVD (OR: 2.51 [1.79–3.52]).

Based on the JNC8, 80.2% (78.9–81.4) of hypertensive individuals were receiving treatment, which decreased to 71.3% (69.9–72.7) when the 2017 ACC/AHA guideline was considered (Table S3). Based on the 2017 ACC/AHA, the ratio of treatment–receiving individuals increased by age (Table S3). The ORs of treatment increased from 2.83 (1.56–5.17) among the 35–44-year-old age group to 13.38 (6.83–26.24) among those ≥75 years old. Treatment percentage increased with insufficient physical activity (OR: 1.21 [1.00–1.47]). Diabetics were more likely than non-diabetics to have received more treatment (OR: 1.79 [1.43–2.24]). Naturally, 92.2% of patients with CVD history were receiving treatment (OR: 3.02 [1.79–5.11]).

The control rate of HTN was 39.1% (37.4–40.7) based on the JNC8. It dropped to 19.6% (18.3–21.0) based on the 2017 ACC/AHA (Table S4). Control significantly decreased from 36.5% among the 25–34-year-old age group (Table S4) to 17.1% among the 55–64-year-old age group (OR: 0.27 [0.09–0.79]). Subsequently, control insignificantly increased among individuals older than 65 years. Control was significantly associated with complementary health insurance coverage (OR: 1.40 [1.06–1.86]). In terms of lifestyle factors, individuals with a lower control were more likely to be obese (OR: 0.56 [0.38–0.82]). Having a CVD history significantly increased HTN control (OR: 2.06 [1.35–3.14]).

Based on the 2017 ACC/AHA, 68.2% of the hypertensive individuals or 37.2% of the entire sample had either BP ≥140/90 or BP ≥130/80 with 10-year ASCVD risk ≥10%, thus were eligible for pharmacologic therapy. Whereas, based on JNC8, 95.7% of hypertensive individuals and 28.6% of the entire sample were eligible for pharmacologic therapy (Table 1). We also found that among participants with BP > 120/80 mmHg, 97.9% were eligible to reduce their salt intake to < 5 g/day, 89.6% consumed insufficient amounts of fruits and vegetables, 69.4% were overweight or obese, and 57.2% were physically inactive.

**Table 1:** Frequency and proportion of participants eligible for pharmacologic and nonpharmacologic therapy*

Type of therapy	%	No.	Sample analysed
Eligible for pharmacologic therapy based on 2017 ACC/AHA			
Among samples	37.2 (36.2–38.2)	9746	26,718
Among hypertensive patients	68.2 (66.9–69.5)	9746	14,147
Eligible for pharmacologic therapy based on JNC8			
Among samples	28.6 (28.0–29.3)	7805	27,165
Among hypertensive patients	95.7 (95.2–96.1)	7805	8148
Nonpharmacologic therapy			
Being overweight or obese	69.4 (68.6–70.2)	11,809	17,028
Insufficient intake of fruits & vegetables (fruits < 2 portions & vegetables < 3 portions)	89.6 (88.9–90.3)	15,527	17,344
Salt Intake > 5 g/day	97.9 (97.6–98.3)	11,718	11,950
Salt Intake > 10 g/day **	42.5 (41.4–43.7)	5208	11,950
Low physical activity (METs < 600/week)	57.2 (56.3–58.2)	8958	15,721
LDL Cholesterol ≥ 130 mg/dL	16.1 (15.3–16.9)	1989	12,248

*Nonpharmacologic therapy is recommended for all individuals with SBP ≥120 mmHg or DBP > 80 mmHg (individuals with elevated, stage 1, and stage 2 hypertension) based on 2017 ACA/AHA

**Since less than 5% of study samples had a salt intake of less than 5 g/day, we considered 10 g/day as the cut-off for the analysis of salt intake

The effects of lifestyle factors on SBP among adults eligible for pharmacologic and non-pharmacologic therapy are presented in Table 2. LDL cholesterol < 130 mg/dL had a large effect size, − 3.56 (− 4.38, − 2.74) mmHg. Just one unit increase in BMI increased SBP by 0.48 (0.42, 0.53) mmHg. Being physically active significantly lowered SBP (− 2.04 (− 2.58, − 1.50)) mmHg. The effect sizes of sufficient intake of fruits and vegetables and salt intake ≥10 g/day were − 1.67 (− 2.49, − 0.86) and 1.52 (0.90, 2.13) mmHg, respectively.

**Table 2:** Predicting factors of blood pressure among participants eligible for pharmacologic and nonpharmacologic therapy

Lifestyle characteristics	Beta (effect size)	95% CI	*P* value
Weight (kg)	0.003*	−0.02, 0.02	0.725
BMI (kg/m^2^)	0.48	0.42, 0.53	< 0.001
Sufficient physical activity ^†^	−2.04	−2.58, −1.50	< 0.001
Intake of fruits & vegetables ^‡^	−1.67	−2.49, −0.86	< 0.001
Salt intake^§^	1.52	0.90–2.13	< 0.001
LDL Cholesterol < 130 mg/dL ^||^	−3.56	−4.38, −2.74	< 0.001
Alcohol consumption ^#^	2.69	1.71, 3.66	< 0.001

*This is the only figure with three decimals in this table

^†^Individuals with sufficient Physical activity (PA) (METs≥600/week) were compared against those with insufficient PA (METs< 600/week)

^‡^Intake of fruits and vegetables was compared between those who consumed sufficient (fruits ≥2 portions & vegetables ≥3 portions) and insufficient portions of fruits and vegetables (fruits < 2 portions & vegetables < 3 portions) in 24 h

^§^Individuals with a salt intake of ≥10 g/day in 24 h were compared against those with an intake of < 10 g/day

^||^Individuals with LDL cholesterol< 130 mg/dL were compared against those with LDL cholesterol ≥130 mg/dL

^#^Non-drinker individuals were compared against drinkers

## Discussion

Switching from JNC8 to the 2017 ACC/AHA created a sharp rise in the prevalence and a drastic decline in awareness, treatment, and control of HTN. Based on the 2017 ACC/AHA, half of the study samples fell into the hypertensive category. Two-thirds of adults in the hypertensive category were unaware, indicating that they were undiagnosed. About one-third of those with awareness remained untreated, and among those treated, less than 20% were under control.

The increase in prevalence upon using the 2017 ACC/AHA guideline in Iran is consistent with a similar increase in prevalence upon using this guideline in other countries e.g. Nepal [21], China [9], and the United States [24, 25]. By adopting the new guideline, the largest increase in prevalence was observed among young and middle-aged individuals, which has also been reported in China [9].

The prevalence and awareness of hypertension in Iran (compared based on the cut-off of 140/90 mmHg) resemble findings reported in other middle-income countries [26, 27]. Despite a higher treatment rate in Iran, the control rate stood at 39.1%, which is yet noticeably lower than Turkey, with 53.9% in 2012, and Lebanon, with 54% in 2014 [28].

Increases in age significantly increased prevalence, awareness, and treatment but decreased the control rate. The likelihood of an increase in prevalence grew by a higher BMI (overweight and obese), higher triglycerides, dyslipidaemia, diabetes, and previous CVD history. Studies also reported a higher prevalence among the overweight, obese [29, 30], diabetics, and those with a history of CVD [31]. Higher awareness and treatment rates were also observed among those with comorbidities of diabetes and CVD [31]. This indicates a higher likelihood of diagnosis and treatment in individuals living with such comorbidities.

Based on the 2017 ACC/AHA, a larger ratio of comorbid patients fell into the hypertensive category (24, 15, 17, and 11% more individuals with dyslipidaemia, high triglycerides, diabetes, and CVD events, respectively). Under this guideline, many high-risk adults might be covered by pharmacologic therapies and be protected against the progression of CVD and diabetic renal diseases [5, 32].

Based on the 2017 ACC/AHA, the proportion of participants with BP > 120/80 mmHg who were eligible for nonpharmacologic therapy was high. 97.9% of participants with BP > 120/80 mmHg need to lower their salt intake to < 5 g/day as recommended by the WHO [33]; 89.6% need to consume sufficient fruits and vegetables, and 69.4% need to lose extra weight.

Though the Tanaka formula provided statistically better estimates for sodium intake in Iran [20], all three common formulas used to estimate sodium intake (Kawasaki, Tanaka, and INTERSALT) were systematically biased with overestimation at lower levels and underestimation at higher levels of sodium intake [34]. In the light of this evidence, we noted that the level of salt intake in Iran was much higher than the 5 g/day cut-off recommended by WHO, therefore even in the presence of underestimation of sodium intake calculation, sodium intake levels exceeded the recommended salt intake level. Thus, the percentage of those eligible for reducing salt intake remained quite large.

Lifestyle factors, LDL cholesterol, physical activity, and BMI had large effect sizes on lowering BP. Based on these findings, non-pharmacologic therapy in hypertensive patients may be considered to modify these lifestyle factors. The modification of these lifestyle factors could be a recommended therapy for low risk adults (ASCVD < 10%) who fall into the hypertensive category under the new guideline.

We contribute to a better understanding of the burden of hypertension based on two distinctive guidelines. Considering the more aggressive cut-off point of 130/90 mmHg resulted in a greater prevalence and lower effective coverage of hypertension. The largest increase in prevalence was observed in adults aged 25–34 years. Given the large population of this age group (16.8 million) in Iran, the number of adults who fall into the hypertensive category remarkably increase from 1.2 million to 5.2 million using the 2017 ACC/AHA. Despite the lowest awareness and treatment rates in this young group, their control rate was highest among all age groups. This implies that targeting younger groups brings about greater benefits for hypertension UHC programs and for the society through maintaining health among the working as well as the reproductive population of the country [35]. Given this potential benefit for Iran, the use of the 2017 ACC/AHA might also benefit other middle-income countries with similar population profiles [9].

Though adopting 2017 ACC/AHA led to a higher prevalence, yet not all adults falling into the hypertensive category were eligible for antihypertensive medications [5]. Based on the 2017 ACC/AHA, 37.2% of Iranian adults aged > 25 years (17.2 million adults) were eligible for pharmacologic therapy and based on JNC8 28.6% (13.4 million adults) of them were [36]. With a minimum unit-cost of treatment around $38 per person [37], pharmacologic therapy would annually cost $653 million and $510 million, respectively, under the 2017 ACC/AHA and JNC8 for all Iranian adults aged > 25 years. Thus, the treatment costs incurred for pharmacotherapy by the health system under the 2017 ACC/AHA guideline was only marginally higher than the treatment costs under the JNC8.

We found that hypertension was very poorly controlled in Iran. Control is by nature a co-creational outcome. Both patient behaviour and an effective structure and process of care play roles in improving this outcome. Based on our findings, patient adherence to a healthy lifestyle and complementary insurance may improve the control rate [29, 30, 38]. We, however, call future research to examine other dimensions of effective structures and processes e.g. evidence-based care plan [39–41] and continuity of care [42, 43] to improve hypertension control.

This research had several limitations, including the challenge of causal inferences from cross-sectional data and potential misclassifications of covariates. We claim no causal relationships as making causal inferences from cross-sectional surveys is challenging. However, some of our criteria do help infer causal relationships; we relied on a compelling theoretical causal model with regards to examining the determining factors for the prevalence, awareness, treatment, and control of hypertension. This was followed by the associations observed between the focal variables as well as holding that the examined covariates and the causes logically precede these four outcomes [44].

We are also aware that there are potential misclassifications of covariates, particularly salt intake and smoking. We classified the participants based on 10 g/day cut-off for salt intake rather than the 5 g/day recommended cut-off point. We did so as a rather small number of our participants had salt intakes of less than 5 g/day.

With regards to our classification of smoking status, we classified never-smokers with former-smokers in the same group, which may affect the magnitude of effects this group has on the outcomes. The health outcomes of a former-smoker might still be influenced by his/her previous smoking history, which may offset the positive effects of the never-smoker on the health outcomes of interest [45]. Furthermore, the effect of smoking status might be incompletely represented by our data choices. We focused on cigarette only and other tobacco products were excluded from our analysis.

The external validity of our findings can be reasonably maintained by the multistage random proportional to size sampling employed. Participants were from all provinces (except one province) and from both urban and rural areas. Given this, the validity of inferences about the identified relationships might be, though not assuredly, maintained over variations in persons or times [46].

## Conclusions

This manuscript applied a more progressive approach toward the measurement of prevalence, awareness, treatment, and control of hypertension services. The prevalence of hypertension markedly increased by the 2017 ACC/AHA guideline and at the same time awareness, treatment, and control sharply declined. Based on the 2017 ACC/AHA, more than half the adults aged ≥25 years became hypertensive, which were mostly represented by the 25–34 age group. Since the control rate among younger adults was higher than among older adults, adopting the 2017 ACC/AHA guideline may benefit the young population of Iran and in the same way other middle-income countries with similar population profiles.

The new guideline lowers the cut-off value for diagnosis and puts a higher proportion of adults in the hypertensive category. Under this guideline, more individuals with high triglycerides, diabetes, and CVD events fell into a hypertensive category. Thus, by adopting the 2017 ACC/AHA a larger proportion of high-risk populations would be eligible for UHC programs. Yet, not all adults falling into a hypertensive category would need or receive antihypertensive medications; a large proportion of them can be treated through lifestyle modifications, based on the effect sizes reported in this study for LDL cholesterol, physical activity, and BMI.

Based on either guideline, Iran has improved the percentage of hypertension treatment; however, the awareness and particularly the control of hypertension remain a challenge. To improve the control rate, efforts should be made to improve both patient behaviour and the quality of healthcare services.

## Supplementary information

**Additional file 1: **Complementary analysis of relationships between daily salt intake and the outcomes. **Table S1.** Prevalence of hypertension based on the 2017 ACC/AHA and JNC8 hypertension guidelines and individual characteristics associated with prevalence according to the 2017 ACC/AHA guideline. **Table S2.** Percentage of hypertension awareness based on the 2017 ACC/AHA and JNC8 hypertension guidelines and individual characteristics associated with awareness according to the 2017 ACC/AHA guideline. **Table S3.** Percentage of hypertension treatment based on the 2017 ACC/AHA and JNC8 hypertension guidelines and individual characteristics associated with treatment according to the 2017 ACC/AHA guideline. **Table S4.** Percentage of hypertension control based on the 2017 ACC/AHA and JNC8 hypertension guidelines and individual characteristics associated with hypertension control according to the 2017 ACC/AHA guideline.

## Data Availability

The datasets analysed during the current study are not publicly available due to national rules and regulations but are available from the corresponding author on reasonable request.

## Abbreviations

2017 ACC/AHA: 2017 ACC/AHA/AAPA/ABC/ACPM/AGS/APhA/ASH/ASPC/NMA/PCNA Guideline for the Prevention, Detection, Evaluation, and Management of High Blood Pressure in Adults
BMI: Body mass index
CVD: Cardiovascular disease
DBP: Diastolic blood pressure
DM: Diabetes mellitus
GPAQ: Global Physical Activity Questionnaire
HbA1c: Glycated haemoglobin (A1c)
HTN: Hypertension
JNC8: The Eighth Joint National Committee on Prevention, Detection, Evaluation, and Treatment (JNC8) guidelines for the Management of High Blood Pressure in Adults
LDL: Low-density lipoprotein
MET: Metabolic equivalents
NIMAD: Iran National Institute for Medical Research Development
OR: Odds Ratio
SBP: Systolic blood pressure
SDG: Sustainable development goal
SES: Socioeconomic status
STEPS: World Health Organization (WHO) STEPwise approach to Surveillance
UHC: Universal Health Coverage
