# Supplementary material for: Insight into blood pressure targets for universal coverage of hypertension services in Iran: the 2017 ACC/AHA versus JNC 8 hypertension guidelines
Source: BMC Public Health. 2020 Mar 17;20:347. doi: 10.1186/s12889-020-8450-1 (PMC7076938; doi:10.1186/s12889-020-8450-1)
Supplement: Supplementary file 1 — Additional file 1: Complementary analysis of relationships between daily salt intake and the outcomes. Table S1. Prevalence of hypertension based on the 2017 ACC/AHA and JNC8 hypertension guidelines and individual characteristics associated with prevalence according to the 2017 ACC/AHA guideline. Table S2. Percentage of hypertension awareness based on the 2017 ACC/AHA and JNC8 hypertension guidelines and individual characteristics associated with awareness according to the 2017 ACC/AHA guideline. Table S3. Percentage of hypertension treatment based on the 2017 ACC/AHA and JNC8 hypertension guidelines and individual characteristics associated with treatment according to the 2017 ACC/AHA guideline. Table S4. Percentage of hypertension control based on the 2017 ACC/AHA and JNC8 hypertension guidelines and individual characteristics associated with hypertension control according to the 2017 ACC/AHA guideline. [file 12889_2020_8450_MOESM1_ESM.docx]

**Additional file 1: Complementary analysis of relationships between daily salt intake and the outcomes.**

The complementary analysis of relationships between daily salt intake and prevalence, awareness, treatment, and control of hypertension is presented in Tables 1-4. In this analysis, salt intake is considered as a continuous variable.

Table 1: Prevalence of hypertension based on the 2017 ACC/AHA and JNC8 hypertension guidelines and individual characteristics associated with prevalence according to the 2017 ACC/AHA guideline

|  | 2017 ACC/AHA | | | | | JNC8 | |
| --- | --- | --- | --- | --- | --- | --- | --- |
|  | %^†^ | 95% CI | OR* | 95% CI | P-value | %^†^ | 95% CI |
| **Overall** | 53.7 | 52.9-54.4 |  |  |  | 29.9 | 29.2-30.6 |
| **Age groups (years old)** |  |  |  |  |  |  |  |
| **25-34** | 30.7 | 29.5-31.9 | 1 (ref) |  |  | 7.3 | 6.7-8.0 |
| **35-44** | 44.8 | 43.5-46.2 | 1.76 | 1.55-2.00 | <0.001 | 16.3 | 15.4-17.4 |
| **45-54** | 60.4 | 59.0-61.8 | 2.86 | 2.49-3.30 | <0.001 | 33.1 | 31.8-34.5 |
| **55-64** | 71.8 | 70.3-73.2 | 4.49 | 3.81-5.29 | <0.001 | 50.6 | 49.0-52.2 |
| **65-74** | 80.2 | 78.5-81.8 | 6.99 | 5.69-8.58 | <0.001 | 64.0 | 62.1-66.0 |
| **≥75** | 82.4 | 80.4-84.3 | 7.97 | 6.29-10.10 | <0.001 | 69.7 | 67.3-72.1 |
| **Gender** |  |  |  |  |  |  |  |
| **Male** | 55.3 | 54.3-56.4 | 1 (ref) |  |  | 27.9 | 27.1-28.8 |
| **Female** | 52.1 | 51.2-53.0 | 0.74 | 0.67-0.82 | <0.001 | 31.7 | 30.9-32.5 |
| **Marital status** |  |  |  |  |  |  |  |
| **Single/divorced/widow** | 53.1 | 51.5-54.6 | 1 (ref) |  |  | 32.2 | 30.7-33.7 |
| **Married** | 53.8 | 53.0-54.6 | 0.90 | 0.80-1.03 | 0.119 | 29.3 | 28.6-30.1 |
| **Area of residence** |  |  |  |  |  |  |  |
| **Urban** | 53.9 | 53.0-54.8 | 1 (ref) |  |  | 29.4 | 28.6-30.2 |
| **Rural** | 52.9 | 51.5-54.3 | 0.89 | 0.80-0.99 | 0.040 | 31.1 | 29.9-32.4 |
| **Wealth status** |  |  |  |  |  |  |  |
| **Poorest** | 54.0 | 52.4-55.6 | 1 (ref) |  |  | 31.8 | 30.3-33.3 |
| **Poor** | 57.6 | 56.0-59.2 | 0.92 | 0.80-1.07 | 0.277 | 35.0 | 33.5-36.5 |
| **Average** | 55.3 | 53.7-56.9 | 0.83 | 0.72-0.97 | 0.017 | 30.8 | 29.4-32.2 |
| **Rich** | 52.0 | 50.3-53.6 | 0.73 | 0.62-0.85 | <0.001 | 27.9 | 26.5-29.3 |
| **Richest** | 50.3 | 48.8-51.9 | 0.69 | 0.58-0.82 | <0.001 | 25.2 | 23.9-26.5 |
| **Years of schooling** |  |  |  |  |  |  |  |
| **No schooling** | 73.7 | 72.2-75.1 | 1 (ref) |  |  | 56.4 | 54.8-58.0 |
| **1-6 years** | 57.6 | 56.3-58.9 | 0.74 | 0.65-0.84 | <0.001 | 34.4 | 33.2-35.7 |
| **7-12 years** | 47.1 | 46.0-48.3 | 0.71 | 0.61-0.83 | <0.001 | 20.6 | 19.7-21.5 |
| **>12years** | 44.2 | 42.6-45.8 | 0.66 | 0.55-0.79 | <0.001 | 19.8 | 18.6-21.1 |
| **Basic health insurance coverage** |  |  |  |  |  |  |  |
| **No** | 50.1 | 47.5-52.6 | 1 (ref) |  |  | 24.3 | 22.1-26.7 |
| **Yes** | 54.0 | 53.2-54.7 | 0.96 | 0.80-1.16 | 0.692 | 30.3 | 29.7-31.0 |
| **Complementary health insurance coverage** |  |  |  |  |  |  |  |
| **No** | 51.7 | 50.9-52.5 | 1 (ref) |  |  | 27.8 | 27.1-28.5 |
| **Yes** | 60.3 | 58.9-61.7 | 1.05 | 0.93-1.19 | 0.394 | 37.2 | 35.8-38.6 |
| **Smoking status** |  |  |  |  |  |  |  |
| **Never-smoker/former-smoker** | 54.0 | 53.2-54.8 | 1 (ref) |  |  | 30.4 | 29.7-31.1 |
| **Daily cigarette-smoker** | 51.1 | 49.1-53.0 | 0.86 | 0.74-1.00 | 0.058 | 25.2 | 23.5-26.9 |
| **Alcohol consumption** |  |  |  |  |  |  |  |
| **No** | 54.2 | 53.4-55.0 | 1 (ref) |  |  | 30.5 | 29.8-31.1 |
| **Yes** | 47.5 | 45.1-49.8 | 1.00 | 0.84-1.19 | 0.969 | 23.3 | 21.3-25.4 |
| **Sufficient intake of fruits & vegetables**  **(fruits≥2 portions & vegetables≥3 portions)** |  |  |  |  |  |  |  |
| **Yes** | 50.9 | 48.8-52.9 | 1 (ref) |  |  | 26.7 | 24.9-28.4 |
| **No** | 54.0 | 53.2-54.8 | 1.06 | 0.92-1.21 | 0.447 | 30.3 | 29.6-31.0 |
| **Sufficient physical activity**  **(metabolic equivalents (MET) ≥ 600/week)** |  |  |  |  |  |  |  |
| **Yes** | 52.4 | 51.3-53.5 | 1 (ref) |  |  | 28.0 | 27.1-29.0 |
| **No** | 55.4 | 54.4-56.4 | 1.02 | 0.93-1.11 | 0.688 | 32.5 | 31.6-33.4 |
| **BMI category** |  |  |  |  |  |  |  |
| **Underweight (<18.5 kg/m^2^)** | 30.7 | 27.5-34.2 | 0.54 | 0.42-0.70 | <0.001 | 14.8 | 12.5-17.4 |
| **Normal weight (18.5–24.9 kg/m^2^)** | 41.8 | 40.6-43.0 | 1 (ref) |  |  | 20.0 | 19.1-20.9 |
| **Overweight (25.0–29.9 kg/m^2^)** | 57.9 | 56.8-59.0 | 1.60 | 1.44-1.77 | <0.001 | 31.1 | 30.1-32.1 |
| **Obesity (≥30.0 kg/m^2^)** | 67.6 | 66.3-68.8 | 2.21 | 1.95-2.49 | <0.001 | 43.8 | 42.4-45.1 |
| **Mean salt intake (gram/day) **** | 9.7 | 9.6-9.7 | 1.01 | 0.99-1.03 | 0.220 |  |  |
| **Dyslipidaemia***** |  |  |  |  |  |  |  |
| **No** | 50.4 | 49.1-51.6 | 1 (ref) |  |  | 28.4 | 27.3-29.5 |
| **Yes** | 60.7 | 59.4-62.0 | 1.15 | 1.05-1.26 | 0.002 | 36.4 | 35.2-37.7 |
| **High triglycerides (fasting triglycerides ≥200 mg/dL)** |  |  |  |  |  |  |  |
| **No** | 53.3 | 52.3-54.3 | 1 (ref) |  |  | 30.6 | 29.7-31.5 |
| **Yes** | 65.9 | 63.6-68.1 | 1.31 | 1.15-1.49 | <0.001 | 40.5 | 38.3-42.8 |
| **Diabetes mellitus****** |  |  |  |  |  |  |  |
| **No** | 51.5 | 50.5-52.5 | 1 (ref) |  |  | 27.6 | 26.7-28.5 |
| **Yes** | 78.1 | 76.0-80.0 | 1.58 | 1.37-1.82 | <0.001 | 60.7 | 58.2-63.1 |
| **Previous CVD events** |  |  |  |  |  |  |  |
| **No** | 53.0 | 52.3-53.8 | 1 (ref) |  |  | 29.0 | 28.3-29.6 |
| **Yes** | 82.5 | 79.0-85.5 | 1.78 | 1.29-2.46+ | 0.001 | 71.5 | 67.4-75.2 |

†This refers to the percentage of hypertension prevalence in each of the sub-categories of individual characteristics. *ORs are estimated for regression analyses with the hypertension cut-off defined by the 2017 ACC/AHA**24-h salt intake is considered as a continuous variable in this analysis. ***Dyslipidaemia refers to either total cholesterol ≥200 mg/dL, high-density lipoprotein (HDL) cholesterol <35 mg/dL, or low-density lipoprotein (LDL) cholesterol ≥130 mg/dL. ****Diabetes mellitus refers to HbA1c >48 mmol/mol or fasting blood sugar (FBS) >126 mg/dL or self-reported diabetes.

Table 2: Percentage of hypertension awareness based on the 2017 ACC/AHA and JNC8 hypertension guidelines and individual characteristics associated with awareness according to the 2017 ACC/AHA guideline

|  | 2017 ACC/AHA | | | | | JNC8 | |
| --- | --- | --- | --- | --- | --- | --- | --- |
|  | %^†^ | 95% CI | OR* | 95% CI | P-value | % ^†^ | 95% CI |
| **Overall** | 37.1 | 36.2-38.0 |  |  |  | 59.2 | 58.0-60.3 |
| **Age groups (years old)** |  |  |  |  |  |  |  |
| **25-34** | 9.7 | 8.4-11.1 | 1 (ref) |  |  | 21.3 | 17.9-25.1 |
| **35-44** | 17.9 | 16.5-19.5 | 1.71 | 1.30-2.24 | <0.001 | 34.7 | 31.5-37.9 |
| **45-54** | 34.0 | 32.3-35.7 | 3.41 | 2.61-4.44 | <0.001 | 54.2 | 51.7-56.6 |
| **55-64** | 51.0 | 49.2-52.8 | 5.92 | 4.51-7.76 | <0.001 | 66.9 | 64.8-68.9 |
| **65-74** | 60.0 | 57.7-62.3 | 9. 92 | 7.33-13.44 | <0.001 | 71.7 | 69.3-74.0 |
| **≥75** | 67.9 | 65.1-70.5 | 13.30 | 9.58-18.46 | <0.001 | 76.8 | 74.0-79.4 |
| **Gender** |  |  |  |  |  |  |  |
| **Male** | 28.9 | 27.8-30.0 | 1 (ref) |  |  | 49.9 | 48.2-51.7 |
| **Female** | 45.1 | 43.9-46.4 | 1.57 | 1.38-1.80 | <0.001 | 66.7 | 65.2-68.1 |
| **Marital status** |  |  |  |  |  |  |  |
| **Single/divorced/widow** | 41.8 | 39.8-43.8 | 1 (ref) |  |  | 63.8 | 61.2-66.3 |
| **Married** | 36.0 | 35.0-37.0 | 1.18 | 0.99-1.41 | 0.060 | 57.9 | 56.6-59.2 |
| **Area of residence** |  |  |  |  |  |  |  |
| **Urban** | 36.6 | 35.5-37.6 | 1 (ref) |  |  | 59.5 | 58.0-60.9 |
| **Rural** | 38.4 | 36.7-40.1 | 1.14 | 0.98-1.32 | 0.081 | 58.5 | 56.3-60.6 |
| **Wealth status** |  |  |  |  |  |  |  |
| **Poorest** | 39.5 | 37.4-41.6 | 1 (ref) |  |  | 60.2 | 57.5-62.8 |
| **Poor** | 40.3 | 38.4-42.3 | 0.86 | 0.71-1.03 | 0.099 | 60.4 | 58.0-62.7 |
| **Average** | 36.4 | 34.5-38.3 | 0.83 | 0.68-1.01 | 0.067 | 58.2 | 55.6-60.8 |
| **Rich** | 35.4 | 33.4-37.4 | 0.97 | 0.79-1.20 | 0.808 | 58.2 | 55.5-60.9 |
| **Richest** | 34.3 | 32.4-36.3 | 1.03 | 0.82-1.30 | 0.786 | 58.8 | 55.8-61.7 |
| **Years of schooling** |  |  |  |  |  |  |  |
| **No schooling** | 57.5 | 55.7-59.4 | 1 (ref) |  |  | 70.8 | 68.8-72.7 |
| **1-6 years** | 39.2 | 37.6-40.8 | 0.77 | 0.65-0.90 | 0.001 | 59.0 | 56.9-61.2 |
| **7-12 years** | 26.6 | 25.2-28.0 | 0.66 | 0.55-0.80 | <0.001 | 49.6 | 47.3-51.9 |
| **>12 years** | 26.6 | 24.6-28.7 | 0.58 | 0.46-0.73 | <0.001 | 51.2 | 47.8-54.5 |
| **Basic health insurance coverage** |  |  |  |  |  |  |  |
| **No** | 25.2 | 22.4-28.3 | 1 (Ref) |  |  | 60.1 | 58.9-61.3 |
| **Yes** | 38.0 | 37.1-38.9 | 1.30 | 0.96-1.75 | 0.088 | 44.4 | 39.4-49.5 |
| **Complementary health insurance coverage** |  |  |  |  |  |  |  |
| **No** | 34.3 | 33.3-35.3 | 1 (ref) |  |  | 56.2 | 54.8-57.6 |
| **Yes** | 45.6 | 43.8-47.4 | 1.27 | 1.10-1.47 | 0.001 | 66.8 | 64.7-68.9 |
| **Smoking status** |  |  |  |  |  |  |  |
| **Never-smoker/former-smoker** | 38.3 | 37.4-39.3 | 1 (ref) |  |  | 60.6 | 59.4-61.8 |
| **Daily cigarette-smoker** | 25.7 | 23.5-28.1 | 0.79 | 0.63-1.00 | 0.046 | 44.6 | 40.9-48.4 |
| **Alcohol consumption** |  |  |  |  |  |  |  |
| **No** | 38.1 | 37.2-39.1 | 1 (ref) |  |  | 60.4 | 59.2-61.6 |
| **Yes** | 24.1 | 21.3-27.0 | 1.03 | 0.78-1.37 | 0.824 | 41.5 | 36.7-46.5 |
| **Sufficient intake of fruits & vegetables**  **(fruits ≥2 portions & vegetables ≥3 portions)** |  |  |  |  |  |  |  |
| **Yes** | 35.7 | 33.0-38.4 | 1 (ref) |  |  | 58.4 | 54.8-61.9 |
| **No** | 37.3 | 36.4-38.2 | 1.09 | 0.89-1.33 | 0.392 | 59.3 | 58.1-60.6 |
| **Sufficient physical activity**  **(metabolic equivalents ≥600/week)** |  |  |  |  |  |  |  |
| **Yes** | 35.4 | 34.0-36.7 | 1 (ref) |  |  | 57.7 | 55.8-59.6 |
| **No** | 40.7 | 39.5-41.9 | 0.98 | 0.87-1.10 | 0.729 | 62.6 | 61.1-64.2 |
| **BMI category** |  |  |  |  |  |  |  |
| **Underweight (<18.5 kg/m^2^)** | 25.3 | 20.4-31.0 | 0.68 | 0.41-1.12 | 0.130 | 47.3 | 38.6-56.1 |
| **Normal weight (18.5–24.9 kg/m^2^)** | 29.2 | 27.7-30.8 | 1 (ref) |  |  | 54.1 | 51.7-56.5 |
| **Overweight (25.0–29.9 kg/m^2^)** | 35.1 | 33.8-36.5 | 1.40 | 1.20-1.63 | <0.001 | 57.7 | 55.8-59.5 |
| **Obesity (≥30.0 kg/m^2^)** | 45.7 | 44.2-47.3 | 1.73 | 1.46-2.06 | <0.001 | 63.3 | 61.4-65.1 |
| **Mean salt intake (gram/day) **** | 9.6 | 9.5-9.7 | 0.96 | 0.94-0.99 | 0.002 |  |  |
| **Dyslipidaemia***** |  |  |  |  |  |  |  |
| **No** | 37.9 | 36.3-39.6 | 1 (ref) |  |  | 59.1 | 56.8-61.3 |
| **Yes** | 39.3 | 37.7-40.9 | 1.02 | 0.90-1.16 | 0.722 | 59.4 | 57.3-61.4 |
| **High triglycerides (fasting triglycerides ≥200 mg/dL)** |  |  |  |  |  |  |  |
| **No** | 38.4 | 37.1-39.7 | 1 (ref) |  |  | 59.5 | 57.8-61.2 |
| **Yes** | 39.2 | 36.5-42.0 | 1.07 | 0.91-1.27 | 0.422 | 58.0 | 54.4-61.5 |
| **Diabetes mellitus****** |  |  |  |  |  |  |  |
| **No** | 33.6 | 32.3-34.9 | 1 (ref) |  |  | 54.5 | 52.6-56.3 |
| **Yes** | 60.0 | 57.2-62.7 | 1.76 | 1.52-2.05 | <0.001 | 73.7 | 70.9-76.3 |
| **Previous CVD events** |  |  |  |  |  |  |  |
| **No** | 35.9 | 35.0-36.8 | 1 (ref) |  |  | 57.9 | 56.7-59.1 |
| **Yes** | 73.5 | 69.2-77.3 | 2.49 | 1.77-3.49 | <0.001 | 83.7 | 79.8-87.0 |

†This refers to the percentage of awareness in each of the sub-categories of individual characteristics. *ORs are estimated for regression analyses with the hypertension cut-off defined by the 2017 ACC/AHA. **24-h salt intake is considered as a continuous variable in this analysis. ***Dyslipidaemia refers to either total cholesterol ≥200 mg/dL, high-density lipoprotein (HDL) cholesterol <35 mg/dL, or low-density lipoprotein (LDL) cholesterol ≥130 mg/dL. ****Diabetes mellitus refers to HbA1c >48 mmol/mol or fasting blood sugar (FBS) >126 mg/dL or self-reported diabetes.

Table 3: Percentage of hypertension treatment based on the 2017 ACC/AHA and JNC8 hypertension guidelines and individual characteristics associated with treatment according to the 2017 ACC/AHA guideline

|  | 2017 ACC/AHA | |  |  |  | JNC8 |  |
| --- | --- | --- | --- | --- | --- | --- | --- |
|  | %^†^ | 95% CI | OR* | 95% CI | P-value | %^†^ | 95% CI |
| **Overall** | 71.3 | 69.9-72.7 |  |  |  | 80.2 | 78.9-81.4 |
| **Age groups (years old)** |  |  |  |  |  |  |  |
| **25-34** | 23.2 | 17.6-29.9 | 1 (ref) |  |  | 44.1 | 34.7-53.9 |
| **35-44** | 43.2 | 38.8-47.7 | 2.83 | 1.55-5.16 | 0.001 | 61.2 | 55.9-66.2 |
| **45-54** | 65.8 | 62.7-68.8 | 5.90 | 3.28-10.58 | <0.001 | 75.4 | 72.4-78.3 |
| **55-64** | 75.4 | 73.0-77.7 | 8.33 | 4.60-15.08 | <0.001 | 81.4 | 79.2-83.5 |
| **65-74** | 82.6 | 80.1-84.9 | 13.61 | 7.18-25.77 | <0.001 | 86.6 | 84.3-88.6 |
| **≥75** | 84.3 | 81.5-86.8 | 13.32 | 6.78-26.16 | <0.001 | 88.2 | 85.6-90.3 |
| **Gender** |  |  |  |  |  |  |  |
| **Male** | 68.3 | 66.2-70.4 | 1 (ref) |  |  | 78.2 | 76.2-80.1 |
| **Female** | 73.1 | 71.4-74.8 | 1.05 | 0.84-1.32 | 0.670 | 81.3 | 79.8-82.8 |
| **Marital status** |  |  |  |  |  |  |  |
| **Single/divorced/widow** | 77.5 | 74.7-80.0 | 1 (ref) |  |  | 83.7 | 81.1-85.9 |
| **Married** | 69.7 | 68.0-71.2 | 0.87 | 0.67-1.14 | 0.325 | 79.3 | 77.8-80.7 |
| **Area of residence** |  |  |  |  |  |  |  |
| **Urban** | 71.6 | 70.0-73.2 | 1 (ref) |  |  | 80.8 | 79.3-82.2 |
| **Rural** | 70.4 | 67.6-73.0 | 1.02 | 0.81-1.28 | 0.889 | 78.7 | 76.2-81.0 |
| **Wealth status** |  |  |  |  |  |  |  |
| **Poorest** | 71.4 | 68.2-74.4 | 1 (ref) |  |  | 79.7 | 76.8-82.3 |
| **Poor** | 75.0 | 72.2-77.6 | 0.82 | 0.62-1.08 | 0.161 | 82.6 | 80.0-84.8 |
| **Average** | 69.8 | 66.6-72.7 | 0.83 | 0.61-1.13 | 0.225 | 78.5 | 75.5-81.1 |
| **Rich** | 69.4 | 66.2-72.5 | 0.84 | 0.60-1.16 | 0.284 | 78.6 | 75.5-81.3 |
| **Richest** | 68.3 | 64.9-71.6 | 1.06 | 0.73-1.54 | 0.766 | 79.7 | 76.5-82.6 |
| **Years of schooling** |  |  |  |  |  |  |  |
| **No schooling** | 78.8 | 76.7-80.7 | 1 (ref) |  |  | 83.7 | 81.7-85.4 |
| **1-6 years** | 71.9 | 69.5-74.1 | 1.03 | 0.80-1.31 | 0.837 | 79.8 | 77.5-81.8 |
| **7-12 years** | 61.4 | 58.4-64.3 | 0.88 | 0.64-1.22 | 0.449 | 75.5 | 72.6-78.2 |
| **>12years** | 67.7 | 63.6-71.6 | 0.93 | 0.64-1.36 | 0.720 | 79.0 | 74.9-82.5 |
| **Basic health insurance coverage** |  |  |  |  |  |  |  |
| **No** | 65.5 | 58.6-71.7 | 1 (ref) |  |  | 76.2 | 69.6-81.7 |
| **Yes** | 71.6 | 70.1-73.0 | 1.12 | 0.70-1.81 | 0.635 | 80.4 | 79.1-81.6 |
| **Complementary health insurance coverage** |  |  |  |  |  |  |  |
| **No** | 69.1 | 67.3-70.8 | 1 (ref) |  |  | 78.3 | 76.7-79.9 |
| **Yes** | 76.3 | 74.0-78.5 | 0.98 | 0.78-1.24 | 0.858 | 84.2 | 82.2-86.1 |
| **Smoking status** |  |  |  |  |  |  |  |
| **Never-smoker/former-smoker** | 72.0 | 70.6-73.4 | 1 (ref) |  |  | 80.8 | 79.5-82.0 |
| **Daily cigarette-smoker** | 62.1 | 56.9-66.9 | 0.96 | 0.65-1.41 | 0.827 | 72.3 | 67.1-77.0 |
| **Alcohol consumption** |  |  |  |  |  |  |  |
| **No** | 72.0 | 70.6-73.4 | 1 (ref) |  |  | 80.8 | 79.5-82.0 |
| **Yes** | 55.7 | 49.2-62.1 | 0.70 | 0.44-1.12 | 0.138 | 65.9 | 58.9-72.2 |
| **Sufficient intake of fruits & vegetables**  **(fruits ≥2 portions & vegetables ≥3 portions)** |  |  |  |  |  |  |  |
| **Yes** | 68.4 | 63.8-72.6 | 1 (ref) |  |  | 79.5 | 75.3-83.1 |
| **No** | 71.5 | 70.1-73.0 | 0.94 | 0.69-1.28 | 0.688 | 80.2 | 78.8-81.5 |
| **Sufficient physical activity**  **(metabolic equivalents (MET) ≥ 600/week)** |  |  |  |  |  |  |  |
| **Yes** | 68.0 | 65.8-70.2 | 1 (ref) |  |  | 77.9 | 75.7-79.8 |
| **No** | 74.9 | 73.2-76.6 | 1.21 | 1.00-1.47 | 0.048 | 83.0 | 81.4-84.4 |
| **BMI level** |  |  |  |  |  |  |  |
| **Underweight (<18.5 kg/m^2^)** | 74.0 | 62.0-83.2 | 0.90 | 0.42-1.93 | 0.785 | 82.4 | 70.6-90.1 |
| **Normal weight (18.5–24.9 kg/m^2^)** | 69.7 | 66.8-72.5 | 1 (ref) |  |  | 78.8 | 76.0-81.4 |
| **Overweight (25.0–29.9 kg/m^2^)** | 69.6 | 67.4-71.7 | 1.00 | 0.77-1.30 | 0.997 | 78.8 | 76.8-80.7 |
| **Obesity (≥30.0 kg/m^2^)** | 72.9 | 70.8-75.0 | 1.31 | 1.00-1.71 | 0.050 | 81.3 | 79.3-83.2 |
| **Mean salt intake (gram/day) **** | 9.6 | 9.5-9.7 | 0.97 | 0.94-1.01 | 0.156 |  |  |
| **Dyslipidaemia***** |  |  |  |  |  |  |  |
| **No** | 70.9 | 68.4-73.3 | 1 (ref) |  |  | 80.8 | 78.6-82.8 |
| **Yes** | 70.7 | 68.2-73.1 | 1.04 | 0.84-1.28 | 0.721 | 77.9 | 75.4-80.2 |
| **High triglycerides (fasting triglycerides ≥200 mg/dL)** |  |  |  |  |  |  |  |
| **No** | 71.5 | 70.0-73.4 | 1 (ref) |  |  | 80.3 | 78.5-82.0 |
| **Yes** | 67.7 | 63.4-71.7 | 0.94 | 0.72-1.22 | 0.632. | 74.5 | 70.1-78.5 |
| **Diabetes mellitus****** |  |  |  |  |  |  |  |
| **No** | 66.3 | 64.1-68.4 | 1 (ref) |  |  | 76.3 | 74.2-78.3 |
| **Yes** | 82.2 | 79.5-84.7 | 1.79 | 1.43-2.24 | <0.001 | 86.1 | 83.4-88.3 |
| **Previous CVD event** |  |  |  |  |  |  |  |
| **No** | 69.8 | 68.3-71.3 | 1 (ref) |  |  | 79.1 | 77.8-80.4 |
| **Yes** | 92.2 | 88.9-94.6 | 3.00 | 1.78-5.08 | <0.001 | 93.4 | 90.1-95.6 |

This refers to the percentage of hypertension treatment in each of the sub-categories of individual characteristics. *ORs are estimated for regression analyses with the hypertension cut-off defined by the 2017 ACC/AHA. **24-h salt intake is considered as a continuous variable in this analysis. ***Dyslipidaemia refers to either total cholesterol ≥200 mg/dL, high-density lipoprotein (HDL) cholesterol <35 mg/dL, or low-density lipoprotein (LDL) cholesterol ≥130 mg/dL. ****Diabetes mellitus refers to HbA1c >48 mmol/mol or fasting blood sugar (FBS) >126 mg/dL or self-reported diabetes.

Table 4: Percentage of hypertension control based on the 2017 ACC/AHA and JNC8 hypertension guidelines and individual characteristics associated with hypertension control according to the 2017 ACC/AHA guideline

|  | 2017 ACC/AHA | |  |  |  | JNC8 |  |
| --- | --- | --- | --- | --- | --- | --- | --- |
|  | % | 95% CI | OR* | 95% CI | P-value | % | 95% CI |
| **Overall** | 19.6 | 18.3-21.0 |  |  |  | 39.1 | 37.4-40.7 |
| **Age groups (years old)** |  |  |  |  |  |  |  |
| **25-34** | 36.5 | 23.8-51.4 | 1 (ref) |  |  | 62.9 | 47.9-75.8 |
| **35-44** | 19.5 | 14.6-25.6 | 0.30 | 0.10-0.91 | 0.033 | 51.4 | 44.4-58.4 |
| **45-54** | 17.8 | 15.0-20.9 | 0.32 | 0.11-0.92 | 0.035 | 40.0 | 36.3-43.8 |
| **55-64** | 17.1 | 15.0-19.5 | 0.27 | 0.09-0.78 | 0.016 | 38.9 | 35.9-42.0 |
| **65-74** | 20.5 | 18.0-23.3 | 0.38 | 0.13-1.09 | 0.071 | 34.5 | 31.4-37.7 |
| **≥75** | 23.0 | 19.9-26.4 | 0.39 | 0.13-1.12 | 0.080 | 39.0 | 35.4-42.8 |
| **Gender** |  |  |  |  |  |  |  |
| **Male** | 20.9 | 18.8-23.2 | 1 (ref) |  |  | 40.9 | 38.2-43.6 |
| **Female** | 18.8 | 17.2-20.5 | 1.14 | 0.85-1.54 | 0.373 | 38.0 | 36.0-40.1 |
| **Marital status** |  |  |  |  |  |  |  |
| **Single/divorced/widow** | 21.3 | 18.5-24.3 | 1 (ref) |  |  | 36.5 | 33.3-39.9 |
| **Married** | 19.1 | 17.7-20.7 | 0.85 | 0.60-1.21 | 0.370 | 39.9 | 38.0-41.8 |
| **Area of residence** |  |  |  |  |  |  |  |
| **Urban** | 20.2 | 18.7-21.9 | 1 (ref) |  |  | 40.7 | 38.8-42.7 |
| **Rural** | 18.0 | 15.6-20.7 | 0.98 | 0.71-1.35 | 0.883 | 35.0 | 32.0-38.2 |
| **Wealth status** |  |  |  |  |  |  |  |
| **Poorest** | 17.9 | 15.2-21.1 | 1 (ref) |  |  | 37.5 | 33.8-41.3 |
| **Poor** | 16.4 | 14.1-19.0 | 0.97 | 0.65-1.43 | 0.868 | 32.7 | 29.6-35.9 |
| **Average** | 18.4 | 15.6-21.6 | 1.01 | 0.64-1.59 | 0.976 | 38.1 | 34.3-41.9 |
| **Rich** | 23.0 | 19.8-26.5 | 1.30 | 0.84-2.02 | 0.233 | 43.8 | 39.9-47.9 |
| **Richest** | 23.7 | 20.3-27.4 | 1.58 | 0.97-2.59 | 0.066 | 45.3 | 41.2-49.5 |
| **Years of schooling** |  |  |  |  |  |  |  |
| **No schooling** | 17.8 | 15.8-20.0 | 1 (ref) |  |  | 33.0 | 30.6-35.6 |
| **1-6 years** | 20.1 | 17.7-22.6 | 1.33 | 0.94-1.87 | 0.104 | 41.2 | 38.2-44.3 |
| **7-12 years** | 20.6 | 17.9-23.6 | 0.92 | 0.60-1.39 | 0.677 | 45.5 | 41.9-49.1 |
| **>12 years** | 22.7 | 18.6-27.5 | 1.25 | 0.76-2.05 | 0.388 | 42.2 | 37.1-47.5 |
| **Basic health insurance coverage** |  |  |  |  |  |  |  |
| **No** | 19.2 | 13.6-26.3 | 1 (ref) |  |  | 37.5 | 29.8-45.8 |
| **Yes** | 19.6 | 18.3-21.1 | 0.64 | 0.32-1.27 | 0.204 | 39.2 | 37.5-40.9 |
| **Complementary health insurance coverage** |  |  |  |  |  |  |  |
| **No** | 18.4 | 16.9-20.1 | 1 (ref) |  |  | 37.3 | 35.3-39.4 |
| **Yes** | 22.5 | 20.0-25.1 | 1.40 | 1.06-1.86 | 0.016 | 43.1 | 40.2-46.1 |
| **Smoking status** |  |  |  |  |  |  |  |
| **Never-smoker/former-smoker** | 19.5 | 18.2-21.0 | 1 (ref) |  |  | 39.0 | 37.3-40.7 |
| **Daily cigarette-smoker** | 21.0 | 16.2-26.8 | 1.04 | 0.57-1.89 | 0.906 | 41.4 | 35.2-47.8 |
| **Alcohol consumption** |  |  |  |  |  |  |  |
| **No** | 19.4 | 18.0-20.8 | 1 (ref) |  |  | 38.8 | 37.1-40.5 |
| **Yes** | 25.7 | 18.9-33.9 | 1.11 | 0.60-2.04 | 0.738 | 47.1 | 38.6-55.9 |
| **Sufficient intake of fruits & vegetables**  **(fruits ≥2 portions & vegetables ≥3 portions)** |  |  |  |  |  |  |  |
| **Yes** | 21.9 | 17.6-27.1 | 1 (ref) |  |  | 44.9 | 39.3-50.6 |
| **No** | 19.3 | 17.9-20.7 | 0.85 | 0.53-1.38 | 0.513 | 38.3 | 36.6-40.1 |
| **Sufficient physical activity**  **(metabolic equivalents (MET) ≥ 600/week)** |  |  |  |  |  |  |  |
| **Yes** | 20.4 | 18.2-22.8 | 1 (ref) |  |  | 41.2 | 38.4-44.1 |
| **No** | 18.9 | 17.3-20.7 | 0.87 | 0.66-1.13 | 0.301 | 37.9 | 35.8-40.0 |
| **BMI level** |  |  |  |  |  |  |  |
| **Underweight (<18.5 kg/m^2^)** | 22.1 | 12.9-35.3 | 0.53 | 0.19-1.50 | 0.232 | 32.6 | 21.3-46.4 |
| **Normal weight (18.5–24.9 kg/m^2^)** | 23.8 | 20.9-27.1 | 1 (ref) |  |  | 42.7 | 39.1-46.4 |
| **Overweight (25.0–29.9 kg/m^2^)** | 20.0 | 17.9-22.3 | 0.73 | 0.52-1.03 | 0.072 | 39.7 | 37.1-42.4 |
| **Obesity (≥30.0 kg/m^2^)** | 16.1 | 14.2-18.3 | 0.56 | 0.38-0.82 | 0.003 | 36.6 | 34.1-39.3 |
| **Mean salt intake (gram) **** | 9.4 | 9.2-9.7 | 0.99 | 0.94-1.05 | 0.746 |  |  |
| **Dyslipidaemia***** |  |  |  |  |  |  |  |
| **No** | 21.1 | 18.3-24.1 | 1 (ref) |  |  | 40.1 | 37.0-43.4 |
| **Yes** | 17.5 | 15.3-19.9 | 0.80 | 0.63-1.01 | 0.064 | 35.8 | 32.9-38.8 |
| **High triglyceride (fasting triglyceride ≥200 mg/dL)** |  |  |  |  |  |  |  |
| **No** | 20.0 | 18.0-22.1 | 1 (ref) |  |  | 38.8 | 36.4-41.2 |
| **Yes** | 16.0 | 12.1-21.0 | 0.86 | 0.63-1.19 | 0.371 | 33.5 | 28.4-39.0 |
| **Diabetes mellitus****** |  |  |  |  |  |  |  |
| **No** | 19.3 | 17.2-21.6 | 1 (ref) |  |  | 38.9 | 36.2-41.6 |
| **Yes** | 19.6 | 16.0-23.7 | 1.01 | 0.76-1.35 | 0.927 | 36.4 | 32.5-40.5 |
| **Previous CVD events** |  |  |  |  |  |  |  |
| **No** | 19.2 | 17.8-20.7 | 1 (ref) |  |  | 38.8 | 37.0-40.5 |
| **Yes** | 23.6 | 19.2-28.7 | 2.05 | 1.34-3.13 | 0.001 | 42.5 | 37.0-48.3 |

†This refers to the percentage of hypertension control in each of the sub-categories of individual characteristics. *ORs are estimated for regression analyses with the hypertension cut-off defined by the 2017 ACC/AHA. **24-h salt intake is considered as a continuous variable in this analysis. ***Dyslipidaemia refers to either total cholesterol ≥200 mg/dL, high-density lipoprotein (HDL) cholesterol <35 mg/dL, or low-density lipoprotein (LDL) cholesterol ≥130 mg/dL. ****Diabetes mellitus refers to HbA1c >48 mmol/mol or fasting blood sugar (FBS) >126 mg/dL or self-reported diabetes.
